# Tilianin suppresses NLRP3 inflammasome activation in myocardial ischemia/reperfusion injury via inhibition of TLR4/NF-κB and NEK7/NLRP3

**DOI:** 10.3389/fphar.2024.1423053

**Published:** 2024-10-23

**Authors:** Suyue Yin, Kaixi Han, Di Wu, Zihan Wang, Ruifang Zheng, Lianhua Fang, Shoubao Wang, Jianguo Xing, Guanhua Du

**Affiliations:** ^1^ Beijing Key Laboratory of Drug Targets Identification and Drug Screening, Institute of Materia Medica, Chinese Academy of Medical Sciences and Peking Union Medical College, Beijing, China; ^2^ Xinjiang Key Laboratory of Uygur Medical Research, Xinjiang Institute of Materia Medica, Urumqi, China

**Keywords:** tilianin, myocardial ischemia-reperfusion injury, NLRP3 inflammasome, NF-κB, NEK7

## Abstract

Tilianin, a flavonoid compound derived from *Dracocephalum moldavica* L., is recognized for its diverse biological functionalities, in particular alleviating myocardial ischemia-reperfusion injury (MIRI). There is ample evidence suggesting that the NLRP3 inflammasome has a significant impact on the development of MIRI. In this study, rats undergoing the ligation and subsequent release of the left anterior descending (LAD) coronary artery and H9c2 cardiomyocytes subjected to oxygen-glucose deprivation/reoxygenation (OGD/R) were used to investigate the effects of tilianin on NLRP3 inflammasome and its anti-MIRI mechanisms. Upon reperfusion, the rats were intraperitoneally injected with tilianin at doses of 3, 10, 30 mg/kg. H9c2 cells were treated with tilianin at concentrations of 10, 30, and 50 μg/mL. Echocardiography, TTC staining and TUNEL staining demonstrated that tilianin remarkably improved cardiac function and mitigated myocardial damage in MIRI rats. Additionally, notable inflammatory response reduction by tilianin was evidenced by subsequent hematatoxylin-eosin (HE) staining, inflammatory cytokines assay, and quantitative proteomics. Further western blotting analysis and immunofluorescence staining showed tilianin decreased the levels of TLR4, p-NF-κB, NLRP3, and ASC in MIRI rats and H9c2 cells exposed to OGD/R, alongside a significant reduction in cleaved gasdermin D, mature IL-1β and IL-18. Molecular docking, cellular thermal shift assay (CETSA) and co-immunoprecipitation (co-IP) assay revealed that tilianin impeded the interaction between NLRP3 and NEK7. Taken together, tilianin protects cardiomyocytes from MIRI by suppressing NLRP3 inflammasome through the inhibition of the TLR4/NF-κB signaling pathway and the disruption of the NEK7/NLRP3 interface. These findings underscore the potential of tilianin as a promising therapeutic candidate for MIRI.

## 1 Introduction

Myocardial infarction (MI), resulting from sudden obstruction of a coronary artery, contributes to an escalating global mortality rate (Klug et al., 2012; Karam et al., 2019). Expedient restoration of blood flow through the epicardial arteries, or alternative reperfusion therapies, during MI’s acute phase presents a significant breakthrough, diminishing immediate fatalities from acute MI (Heusch, 2020; Algoet et al., 2023). Regrettably, reperfusion subsequently induces additional cardiac damage, a paradoxical phenomenon tied to a detrimental condition called myocardial ischemia/reperfusion injury (MIRI). MIRI accounts for up to 50% of the final size of myocardial infarction and involves the regulation of multiple cell death modes, activation of inflammatory responses, generation of oxygen free radicals, and calcium overload (Yellon and Hausenloy, 2007; Konstantinidis et al., 2012; Koshinuma et al., 2014).

Tilianin is a flavonoid glycoside extracted from *Dracocephalum moldavica* L. (*D. moldavica*) which is prevalently employed in traditional Chinese medicine therapies (Aćimović et al., 2022). This compound exhibits exceptional anti-inflammatory, antioxidant, and anti-tumor effects (Shen et al., 2019; Jiang et al., 2020b; Li and Xu, 2022). In addition, tilianin has been demonstrated to have significant protective effects in I/R injury, particularly by suppressing inflammatory responses (Jiang et al., 2019; Liu Z. et al., 2022). However, its underlying mechanism remains unclear, limiting its extended clinical applications.

Extensive evidence implies that the NLRP3 inflammasome plays a critical role in MIRI pathogenesis (Qiu et al., 2017; Zhang et al., 2020). The NLRP3 inflammasome, located in the cytoplasm, is a multiprotein heteromeric complex consisting of intracellular receptor NLRP3, ASC, and the downstream protease caspase-1 (Huang et al., 2021; Leu et al., 2023). It has been stated that NLRP3 inflammasome activation entails two critical stages: priming and activation (Paik et al., 2021). The priming phase involves the induction of NLRP3 expressions in response to various stimuli, with the engagement of TLR4 and the phosphorylation of NF-κB (Chen et al., 2022; Dong et al., 2022). Consequent post-translational modifications enable NLRP3 to conform aptly for self-oligomerization and interaction with ASC, facilitating inflammasome activation (Gritsenko et al., 2020). Aberrant NLRP3 inflammasome activation contributes to the progression of many diseases, including type 2 diabetes, cancers, neurodegenerative diseases, and MIRI (Péladeau and Sandhu, 2021; Sharma and Kanneganti, 2021).

In this study, rats exposed to LAD ligation and H9c2 cells subjected to oxygen-glucose deprivation/reoxygenation were applied to ascertain the potential protective effects of tilianin and its impact on the NLRP3 inflammasome. The results inferred that tilianin significantly inhibited the NLRP3 inflammasome by suppressing TLR4/NF-κB and disrupting the interactions between NLRP3 and NEK7.

## 2 Materials and methods

### 2.1 Chemicals and reagents

**FIGURE 1:**
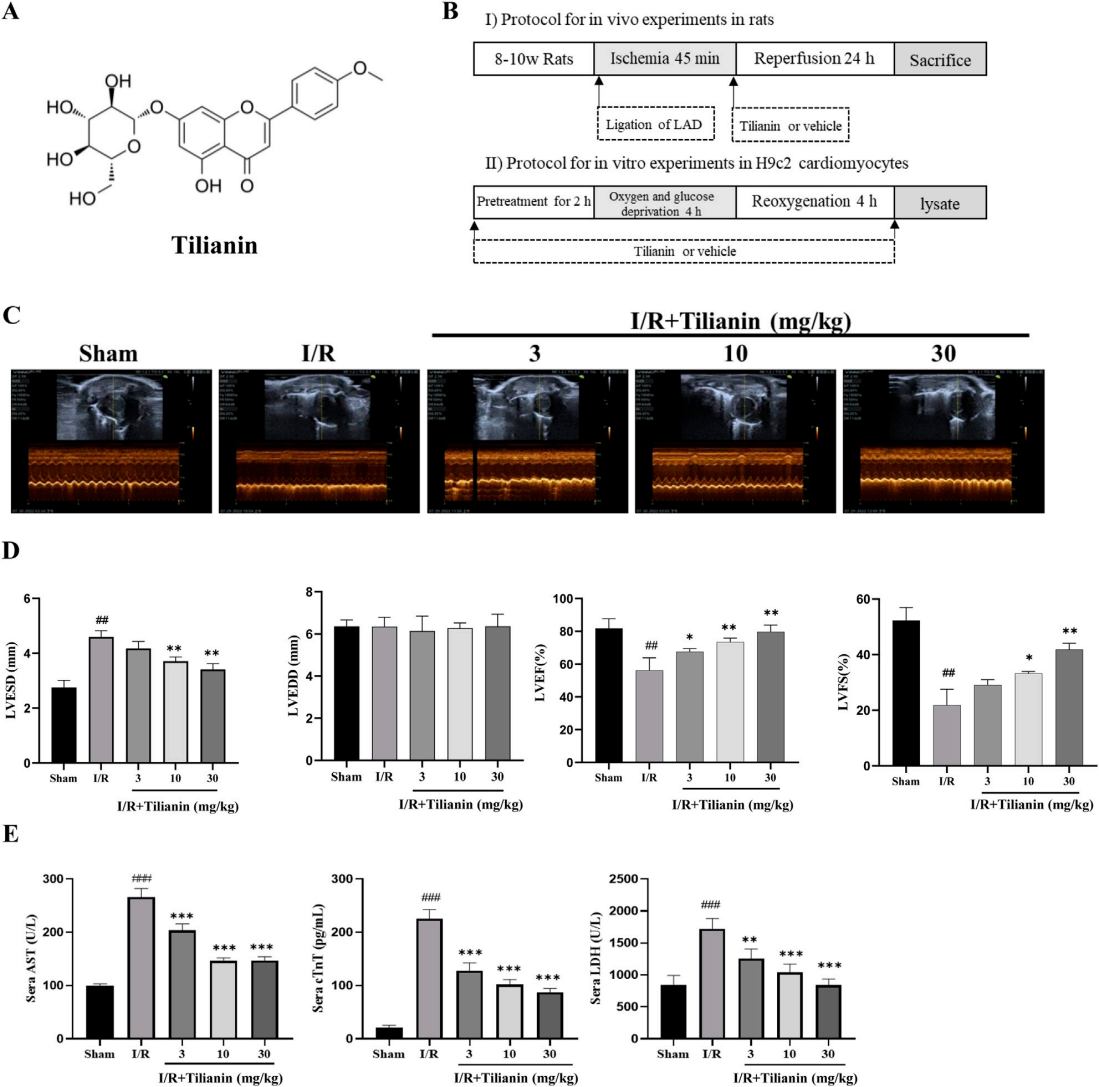
Tilianin improved cardiac function and alleviated myocardial damage in rats subjected to MIRI. **(A)** Chemical structure of tilianin; **(B)** Schematic diagram of experimental protocol; **(C, D)** LVFS, LVEF, LVESD, and LVEDD were estimated from short-axis M-mode tracing of the left ventricle; **(E)** Tilianin suppressed the release of serum AST, cTnT and LDH in rats undergoing MIRI. n = 6 per group. ##*P* < 0.01, ###*P* < 0.005 vs. sham. *P < 0.05, **P < 0.01 and ***P <0.005 vs. I/R group.

Tilianin (purity > 97%) was provided by Xinjiang Institute of Materia Medica. Dimethyl sulfoxide (DMSO) was purchased from Sigma (ST Louis, MO, United States). The chemical structure of tilianin is shown in Figure 1A. For the *in vivo* and *in vitro* treatment, tilianin was dissolved in DMSO and then diluted in phosphate buffer solution (PBS). Other chemicals were of no specific analytical grade.

### 2.2 Animals

Male Sprague-Dawley rats (250–280 g) were obtained from Beijing Vital River Laboratory Animal Technology (Beijing, China), housed under controlled environmental conditions (a normal light-dark cycle, 22°C), and fed commercial food and water in the animal care facility of our institute. All experimental procedures were permitted by the Institute of Materia Medica’s Animal Care and Use Committee on the Ethics of Animal Experiments.

### 2.3 Establishment of MIRI in rats

Myocardial infarction/reperfusion was induced in SD rats by the ligation of the left anterior descending coronary artery (LAD) as shown in our previous study (Wang D. S. et al., 2020). Rats were immobilized in a supine position on a heating pad and anesthetized with 2% isoflurane by inhalation through a mask. The heart was popped out following a thoracotomy along the left fourth intercostal space. A 6–0 suture thread with the needle was tied into a slipknot to ligate the LAD at 3 mm below the extension line of the inferior edge of the left auricle of the heart. All animals that underwent I/R surgery or sham surgery were divided into 5 groups (n = 10 per group): Sham rats, I/R rats, and I/R rats treated with tilianin at doses of 3, 10, or 30 mg/kg 45 min of ischemia and 24 h of reperfusion were carried out. Rats were intraperitoneally injected with vehicle or tilianin when reperfusion started. The protocol is shown in Figure 1B.

### 2.4 Echocardiography

Transthoracic echocardiography was performed on anesthetized rats (with 2% isoflurane) by using a VINNO 6 LAB ultrasonic imaging system (VINNO, Suzhou, China). The heart image was captured in two-dimensional mode in the parasternal short-axis view. M-mode tracings were recorded at the papillary muscle level and the following parameters were measured: left ventricular end-systolic diameter (LVESD), left ventricular end-diastolic diameter (LVEDD), ejection fraction (LVEF), and fractional shortening (LVFS).

### 2.5 Measurement of inflammatory cytokines and cardiac markers in sera

After echocardiography, the anesthetized rats were fixed on a dissection table, the abdomen was opened to expose the abdominal aorta, and blood was collected from the abdominal aorta with a disposable blood collection needle. The samples were allowed to clot naturally for 30–60 min at room temperature without shaking or vibration. Centrifuged the samples at 4°C, 5,000 rpm for 15 min (Allegra TMX-22R Centrifuge, Beckman, Germany) to collect the supernatant. The levels of lactate dehydrogenase (LDH) and aspartate transaminase (AST) were detected via commercial kits (Bio-Technology and Science, Beijing, China) based on the spectrophotometric assays. Cardiac troponin T (cTnT), IL-1β, and tumor necrosis factor-alpha (TNFα) were tested by commercial enzyme-linked immunosorbent assay (ELISA) kits (Cusabio Technology, Wuhan, China).

### 2.6 Evans blue and 2,3,5-triphenyl-tetrazolium chloride (TTC) double-staining

Evans blue and TTC double-staining was conducted as the previous study (Wang D. S. et al., 2020). Before harvesting the heart, carefully cannulate the proximal end of aorta and then slowly injected 1% Evans Blue solution, until the rat heart rapidly turned blue beyond the ligation line. The hearts were quickly excised, frozen, and sliced the heart into 5–6 sections at 2 mm thickness. Stained the slices in 1% TTC solution (diluted in PBS with pH 7.4) at 37°C for 15 min and photographed. The infarct area (IF, strained red), area at risk (AAR, strained blue) and the whole left ventricle (LV) were calculated using ImageJ software (NIH, Bethesda, United States). The infarct size was represented by the ratio of IF/AAR.

### 2.7 HE staining

HE staining protocol was carried out as the description of previous study (Li et al., 2018). The heart samples were washed immediately with iced saline and fixed in 4% buffered paraformaldehyde solution at room temperature for at least 12 h and embedded in paraffin. After dewaxing with xylene and hydration with different concentrations of ethanol, the sections were stained with hematoxylin and eosin and sealed with neutral gum. Images were acquired and analyzed via Case Viewer (3DHISTECH, Budapest, Hungary). Semi-quantification of inflammatory cells was obtained from 6 random fields in cardiac tissues using ImageJ.

### 2.8 Quantitative proteomics and differential protein abundance analysis

Proteomic analysis based on the label-free proteome quantification was performed on the left ventricular tissue from rats subjected to I/R or I/R + Tilainin 30 mg/kg group. The main procedures included protein extraction and digestion, nano-UPLC separation, LC-MS/MS analysis, and data analysis. Raw data were processed by MaxQuant (2.0.1.0). The protein database, obtained from the UNIPROT database (uniprot-proteome-rat-2021.2), was used for MaxQuant search libraries. The quantification type was non-labelled quantification (nLFQ) including match between runs. nLFQ quantification results were log transformed, and missing values were filled using Perseus software for random sampling from a normal distribution. Subsequently, the statistical analysis of normalized quantitative results was performed to obtain the differentially expressed proteins. The proteins with fold change >1.2 or <0.83, *P* value ≥0.05, and unique peptides ≥2 were defined as significantly different and subjected to subsequent GO and KEGG pathway analysis.

### 2.9 TUNEL staining assay

TUNEL staining was performed with a commercial kit (Roche, Mannheim, Germany) according to the manufacturer’s instructions. TUNEL-positive nuclei were counted in 10 random high-magnification fields (400×) of each section. The apoptosis intensity was expressed as the percentage of TUNEL-positive cells relative to the total cells (DAPI-positive cells).

### 2.10 Cell culture and treatment

H9c2 cardiomyocytes were obtained from Sciencell Research Laboratories (Carlsbad, United States). Cells were cultured in Dulbecco’s Modified Eagle Medium (DMEM; Gibco, New York, United States) supplemented with 10% fetal bovine serum (Procell, Wuhan, China) and incubated at 37°C in a humidified atmosphere containing 5% CO_2_.

The oxygen-glucose deprivation/reoxygenation procedures were carried out according to previous studies with a slight modification (Figure 1B) (Liu T. et al., 2022). Briefly, H9c2 cells were cultured in PBS for 2 h to induce OGD and then replaced the supernatant with a complete medium and incubated for 4 h to mimic reoxygenation. H9c2 cells were seeded at 8,000 cells/well in a 96-well plate for 24 h. And then H9c2 cells were randomly divided into two groups with or without OGD/R. OGD/R groups were treated with tilianin at 0, 1, 3, 10, 30, and 100 μg/mL respectively from 2 h before OGD/R to its end. Cell viability was assessed using CCK-8 on a FlexStation 3 Multi-Mode Microplate Reader (Molecular Devices, San Jose, United States).

### 2.11 Western blot analysis

Heart tissues or collected cells were lyzed in RIPA lysis buffer (Applygen, C1053, China) supplemented with a protease inhibitor cocktail (Applygen, P1265, China) and a phosphatase inhibitor cocktail (Applygen, P1260, China). Before SDS-PAGE (Bio-Rad, Hercules, United States), the supernatant was analyzed by BCA assay (Applygen, P1511, China) to quantify the protein concentration. SDS-PAGE was performed by loading equal amounts of the proteins into the gel, which were then transferred to PVDF membranes (Merck Millipore, United States). The membrane was blocked in 5% BSA solution. Incubated the target proteins at 4°C overnight with the following corresponding primary antibodies: NLRP3 (CST, United States, Cat# 15101), cleaved caspase-1 (CST, United States, Cat#89332), ASC (Santa Cruze, United States, Cat# sc514414), IL-1β (CST, United States, Cat#12242), IL-18 (Abcam, United States, Cat# ab191860), NF-κB p65 (CST, United States, Cat# 8242), phosphorylated NF-κB p65 (CST, United States, Cat#3033), GAPDH (ImmunoWay, China, Cat# YM3029), β-actin (CST, United States, Cat# 3700), cleaved GSDMD (CST, United States, Cat# 36425), and NEK7 (Abcam, United States, Cat# ab133514). Then the bands were incubated with horseradish peroxidase-conjugated anti-rabbit (Gene-Protein Link, China, Cat# P03S02M) or anti-mouse (Gene-Protein Link, China, Cat# P03S01M) secondary antibodies for 2 h at room temperature. The blots were visualized using ECL kits (Invitrogen, United States) and analyzed by ImageJ software.

### 2.12 Immunofluorescence staining

H9c2 cells were seeded on 6 well cell culture plates. After OGD/R and tilianin treatment, cells were fixed with 4% paraformaldehyde, permeabilized in PBS containing 0.3% Triton X-100, and blocked in 0.5% bovine serum albumin (BSA). Next, the cells were incubated with anti-ASC antibody (diluted 1:250; Santa Cruz, United States, Cat# sc514414) in combination with goat anti-mouse secondary antibody conjugated with Alexa Fluor 488 (diluted 1:1,000; CST, United States, Cat# 4408S) in 1% BSA-PBS; Nuclei were counterstained with DAPI (Solarbio, China, Cat# C0065). Fluorescent images were captured under a fluorescence microscope (Nikon ECLIPSE Ti, Shanghai, China).

### 2.13 Co-immunoprecipitation (co-IP) assay

The procedures of co-IP were carried out in accordance with the instructions of Protein A (or A/G) Immunoprecipitation Kit (Beaver, China, Cat#22202). Cell lysates extracted from H9c2 were incubated with anti-NLRP3 antibody at 4°C for overnight, followed by the addition of Protein A/G plus agarose and incubation with slow shaking. After centrifugation and washing, the immunoprecipitated complex was subjected to Western blot analysis, employing both anti-NEK7 and anti-NLRP3 antibodies.

### 2.14 Molecular docking

CDOCKER and ZDOCK modules in Discovery Studio 2018 (BIOVIA, United States) with previously reported protocol was used to reveal the binding modes of tilianin/NEK7 and NEK7/NLRP3 respectively (Liu et al., 2023). The 2D chemical structure of tilianin was obtained from Pubchem to carry out the docking simulation. The crystal structure of NEK7 with a resolution of 4.04 Å (PDB ID: 6S76) was downloaded from the Protein Data Bank (https://www.rcsb.org/ accessed on 27 February 2024). Before docking, remove ligand molecules from the NEK7 crystal structure, define the binding site, and then reconnected back to the pre-defined active sites. Finally, the values of docking energy between tilianin and NEK7 were calculated and compared with that of the original ligand (Diethylene glycol) and NEK7. To further test the influence of tilianin on the docking modes of NEK7 and NLRP3, the ZDOCK algorithm was used to assess the interactions and affinity of tilianin/NEK7 complex and NLRP3. The tilianin/NEK7 complex with the lowest docking energy was selected for further docking with NLRP3. Using a 15°angular step size, 5,400 poses were generated and ranked, of which the top 100 were clustered. Then the interface of proteins was analyzed, and the interactions results were compared with the NEK7/NLRP3 complex.

### 2.15 CETSA assay

CETSA assay was carried out as the previous studies with slight modifications (Jafari et al., 2014; Krishnamachary et al., 2019). H9c2 cells were cultured in DMEM supplemented with 10% fetal bovine serum, and incubated at 37°C in a humidified atmosphere containing 5% CO_2_ for 24 h. Subsequently, 30 μg/mL tilianin or an equivalent volume of vehicle (0.1% DMSO) was administered to the cells for an additional 24 h. The cell lysates were prepared using two freeze-thaw cycles and were evenly divided into six tubes, which were heated at 40, 45, 50, 55, 60, and 65°C for 5 min, respectively. The supernatants were collected after centrifugation at 20,000 g at 4°C for 20 min. Each supernatant was then combined with 5× loading buffer and heated at 70°C for 10 min to prepare for Western blot analysis.

### 2.16 Statistical analysis

The data were presented as mean ± SD of each group. Significant differences among the means were analyzed with GraphPad Prism 8 software (GraphPad Software, La Jolla, CA, United States) using ordinary one-way analysis of variance (ANOVA) followed by Dunnett’s *post hoc* test. The differences with *P*-values of less than 0.05 were considered statistically meaningful.

## 3 Results

### 3.1 Tilianin improved cardiac function in rats undergoing MIRI

Echocardiography was used to investigate whether tilianin improves heart function in rats undergoing MIRI (Figure 1C). Compared to sham rats, LVESD in I/R rats increased significantly, while LVEDD showed no substantial change. LVEF and LVFS decreased (Figure 1D). Treatment with tilianin at doses of 10 and 30 mg/kg reduced LVESD and improved LVEF and LVFS compared to I/R rats, without a significant effect on LVEDD. In contrast to the sham group, serum levels of AST, cTnT and LDH were significantly elevated following I/R injury in I/R rats. However, treatment of tilianin at doses of 3, 10 and 30 mg/kg profoundly ameliorated serum levels of these indicators, as shown as Figure 1E.

### 3.2 Tilianin reduced infarct size and apoptosis in rats undergoing MIRI

**FIGURE 2:**
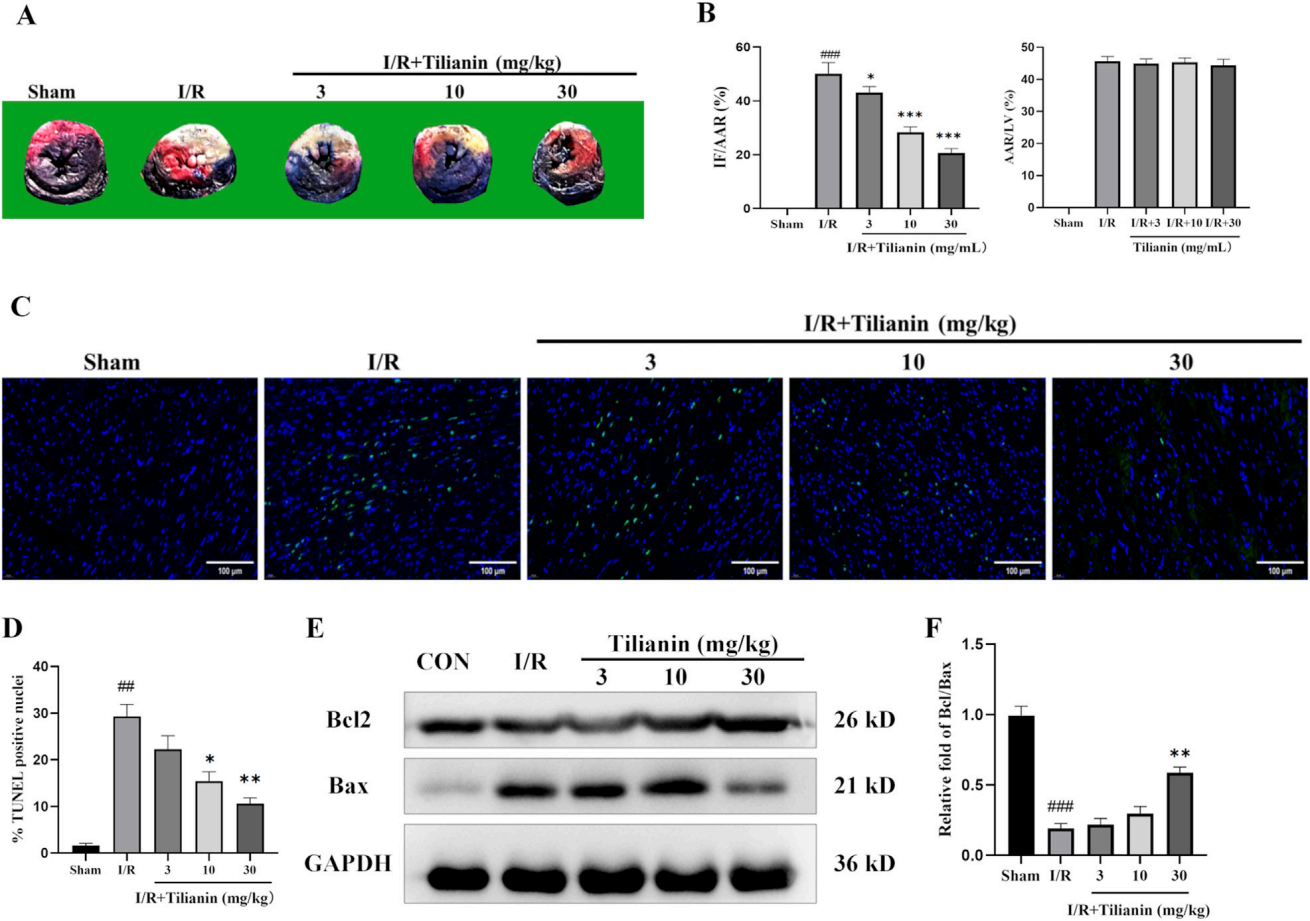
Tilianin reduced infarct size and apoptosis in rats undergoing MIRI. **(A)** Representative images of heart sections using double-staining of Evans blue and TTC, and the percentages of IF/AAR and AAR/LV were shown as **(B)**. **(C, D)** TUNEL staining indicated the percentages of cardiomyocytes stained positive for apoptosis. TUNEL-positive nuclei were stained with green, and the total nuclei were stained with blue; Scale bar, 50 μm. n = 3 per group. **(E, F)** The expressions of Bax and Bcl2 in the left ventricular tissue were determined by western blotting, and GAPDH served as the loading control. n = 4 per group. ##*P* < 0.01, ###*P* < 0.005 vs. sham. *P < 0.05, **P < 0.01 vs. I/R group.

Double staining of Evans blue and TTC demonstrated that tilianin remarkably reduced the percentage of IF/AAR in rats after MIRI when compared with the I/R group, whilst the percentage of AAR/LV showed no difference (Figures 2A, B). Moreover, TUNEL staining showed that myocardial ischemia injury raised the percentage of TUNEL-positive cardiomyocytes (Figures 2C, D). In contrast, tilianin significantly decreased apoptosis. Western blotting results further confirmed that tilianin could increase the ratio of Bcl-2/Bax during MIRI compared with the I/R group (Figures 2E, F).

### 3.3 Tilianin attenuated the inflammation in rats undergoing MIRI

**FIGURE 3:**
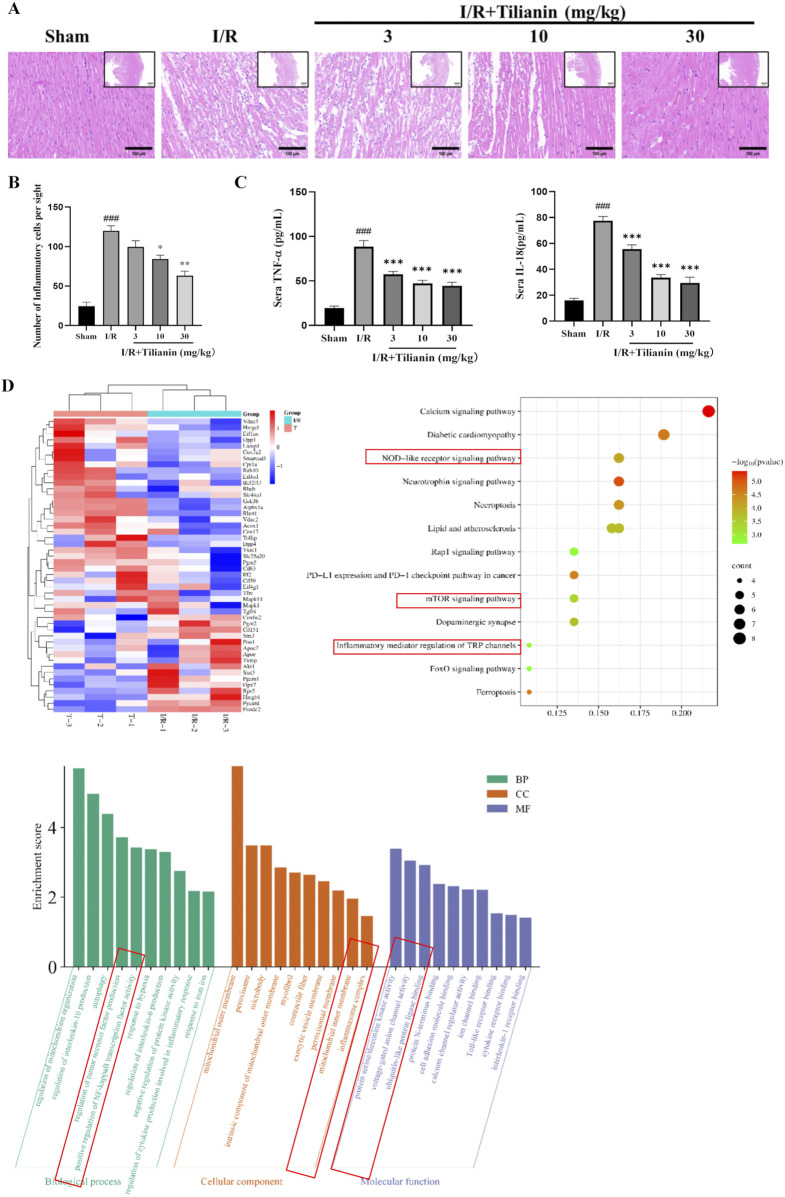
Tilianin attenuated the inflammatory responses in rats undergoing MIRI. **(A, B)** Representative images of H&E staining showed the injury of left ventricular cardiomyocytes and the infiltration of inflammatory cells, Scale bar, 100 μm (large), 500 μm (small). n = 4 per group. **(C)** Tilianin suppressed the release of IL-18 and TNF-α. n = 6 per group. **(D)** Proteomic analysis of interventricular septum from I/R (I/R, green) and I/R + tilianin 30 mg/kg group (T, red) rats. ###*P* < 0.005 vs. sham. *P < 0.05, **P < 0.01, ***P < 0.005 vs. I/R group.

Hematoxylin and Eosin staining showed severe inflammation infiltration among cardiomyocytes in the I/R group (Figure 3A), but tilianin administration at 10 and 30 mg/kg reduced the number of inflammatory cells to approximately half of that was observed in the I/R group (Figure 3B). I/R surgery significantly elevated serum levels of IL-18 and TNF-α while tilianin treatment effectively reduced their levels (Figure 3C). In addition, proteomics analysis identified 51 differentially expressed proteins between I/R group and I/R + Tilianin group, which was presented as heat map (Figure 3D). Moreover, Gene Ontology (GO) and Kyoto Encyclopedia of Genes and Genomes (KEGG) enrichment analysis showed that the differentially expressed genes were predominantly involved in inflammation-related signaling pathways, including the NOD-like receptor and mTOR signaling pathways.

### 3.4 Tilianin diminished phosphorylation of NF-κB and activation of NLRP3 inflammasome in rats undergoing MIRI

**FIGURE 4:**
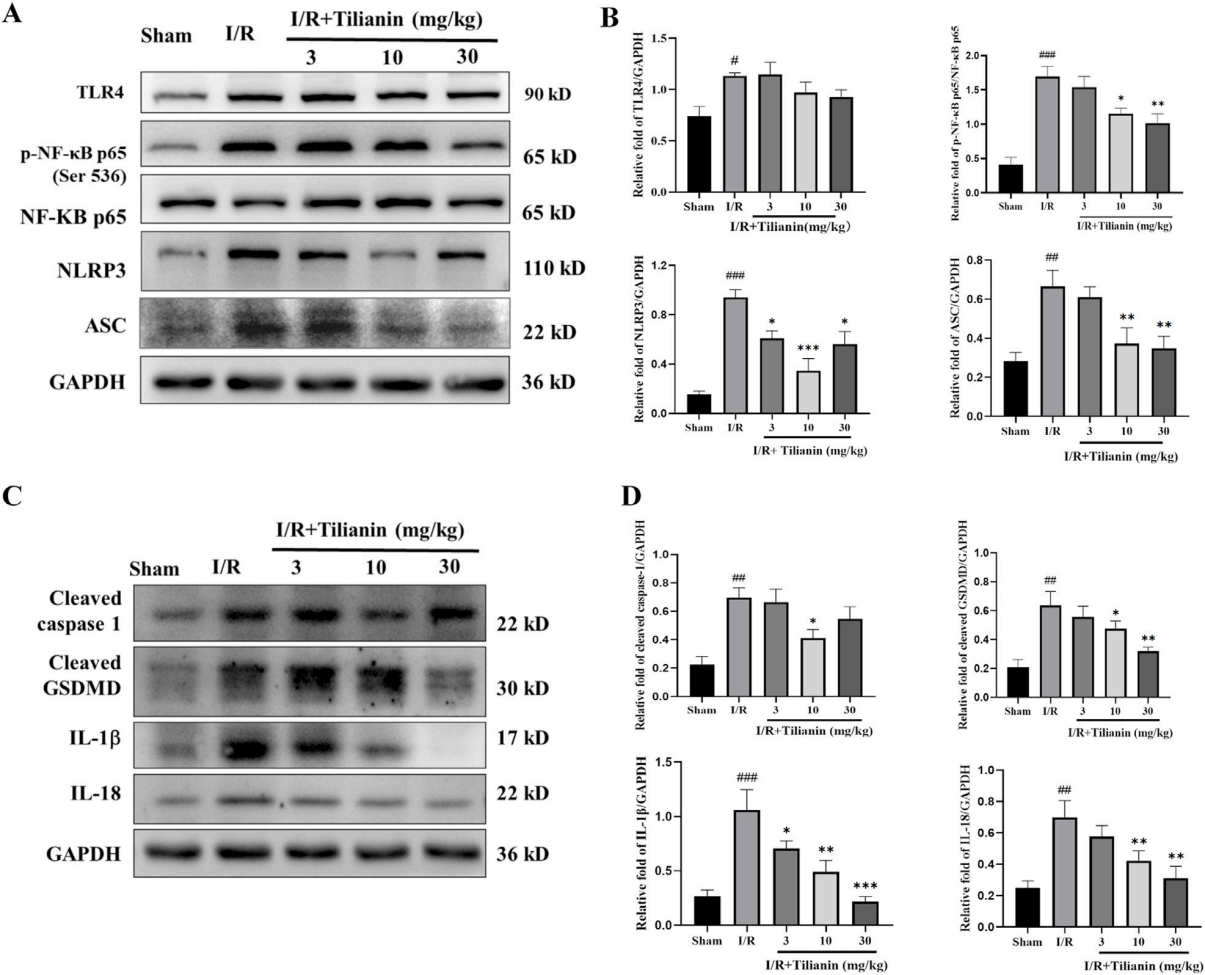
Tilianin reduced NF-κB phosphorylation and NLRP3 inflammasome activation in rats undergoing MIRI. **(A, B)** The levels of TLR4, p-NF-κB, NF-κB, NLRP3, and ASC in left ventricular tissue were determined by western blot. **(C, D)** Representative western blot bands of cleaved caspase-1, cleaved GSDMD, IL-1β, and IL-18 in left ventricular tissue. n = 4 per group. #*P* < 0.05, ##*P* < 0.01, ###*P* < 0.005 vs. sham. *P < 0.05, **P < 0.01, ***P < 0.005 vs. I/R group.

The results delineated above suggested that tilianin’s efficacy in alleviating MIRI is closely linked to the inhibition of inflammation. To delve deeper into the underlying mechanism, we firstly investigated the levels of p-NF-κB and NLRP3 (Figures 4A, B). Western blotting results showed that the elevated levels of TLR4, p-NF-κB, NLRP3, and ASC in the I/R group compared to the sham group. Conversely, tilianin administration markedly diminished the phosphorylation of NF-κB p65 and the level of NLRP3. Moreover, tilianin notably reverted the upregulated levels of cleaved caspase-1 and cleaved GSDMD, and attenuated IL-18 and IL-1β secretion in rats subjected to MIRI (Figures 4C, D). These observations underscored that tilianin’s protective effects might be attributed to the suppression of NLRP3 inflammasome.

### 3.5 Tilianin suppressed NLRP3 inflammasome activation in H9c2 cells subjected to OGD/R

**FIGURE 5:**
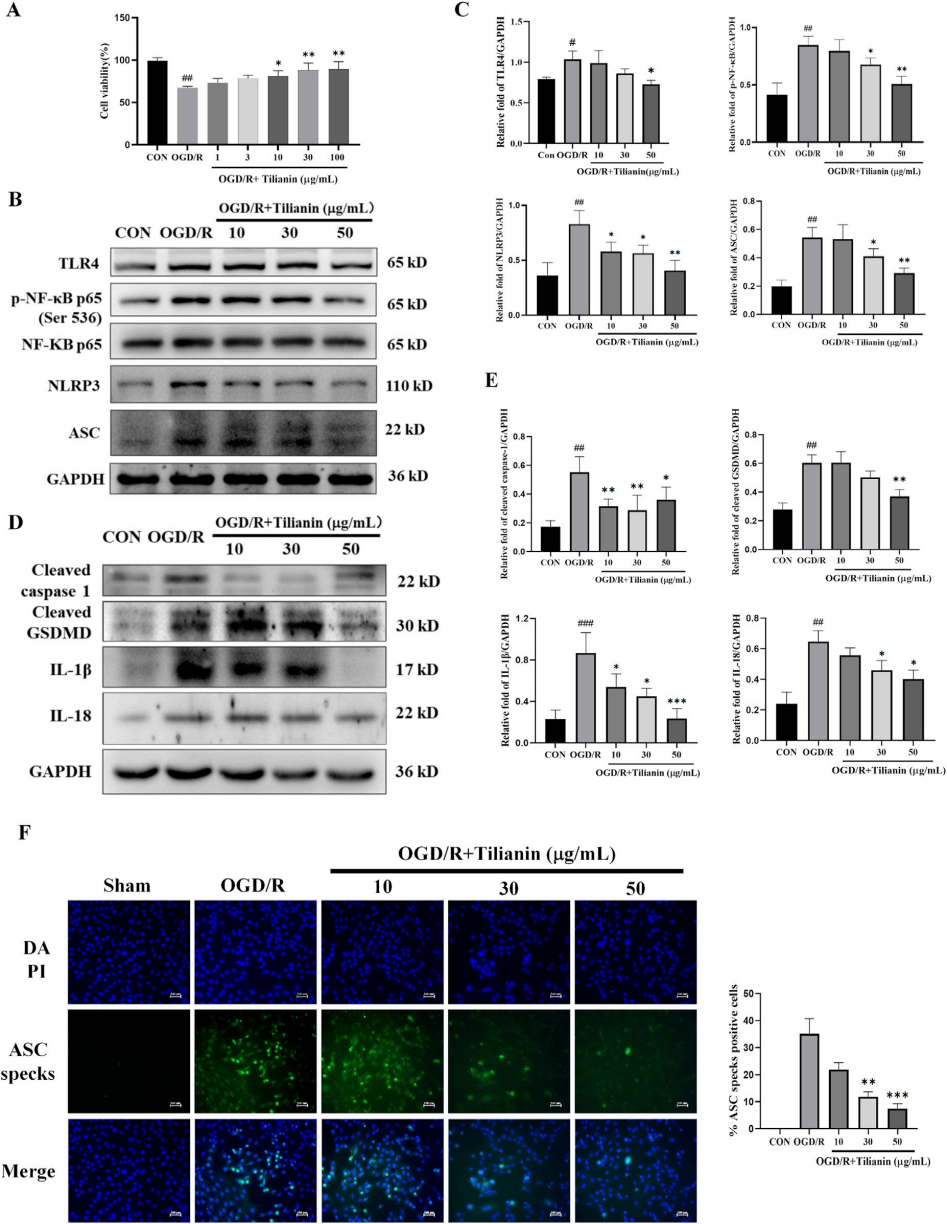
Tilianin suppressed the activation of NLRP3 inflammasome in OGD/R-treated H9c2 cells. **(A)** Tilianin protected H9c2 cardiomyocytes against OGD/R-induced cytotoxicity. **(B, C)** The levels of TLR4, p-NF-κB, NF-κB, NLRP3, and ASC in H9c2 cells. **(D, E)** Representative western blot bands and results of cleaved GSDMD, IL-1β, IL-18, and cleaved caspase-1 in H9c2 undergoing OGD/R. n = 4 per group. **(F)** Tilianin reduced ASC specks production in H9c2 cells with OGD/R. n = 3 per group. #*P* < 0.05, ##*P* < 0.01, ###*P* < 0.001 vs. Control. *P < 0.05, **P < 0.01, ***P < 0.001 vs. OGD/R group.

To further elucidate the effects of tilianin on inhibiting NLRP3 inflammasome activation, we established an H9c2 cellular oxygen-glucose deprivation/reperfusion model to simulate the myocardial ischemia-reperfusion injury. The effect of tilianin on OGD/R-treated H9c2 cells was examined by cell viability. The results of the CCK8 assay demonstrated that H9c2 cell viability declined to approximately 65% compared to the control group after experiencing oxygen and glucose deprivation for 4 h and reoxygenation for 4 h (Figure 5A), whereas the administration of tilianin at doses of 10–100 μg/mL notably enhanced the viability of H9c2 cells exposed to OGD/R injury. Given that the molecular weight of tilianin and higher molar concentration, subsequent *in vitro* experiments utilized concentrations of 10, 30, and 50 μg/mL. Consistent with the findings in the MIRI rats, tilianin reduced levels of TLR4, NF-κB phosphorylation, NLRP3 and ASC in H9c2 cells subjected to OGD/R. Notably, the downregulation of cleaved caspase-1, cleaved GSDMD, IL-1β, and IL-18 also underscored that tilianin’s potent inhibitory effect on NLRP3 inflammasome (Figures 5B, C). Immunofluorescence staining results further supported these findings that tilianin markedly suppressed the increase of ASC specks induced by OGD/R, indicative of decreased NLRP3 inflammasomes activation (Figures 5D, E).

### 3.6 Tilianin lowered the level of NEK7 and disrupted the interaction of NLRP3/NEK7

**FIGURE 6:**
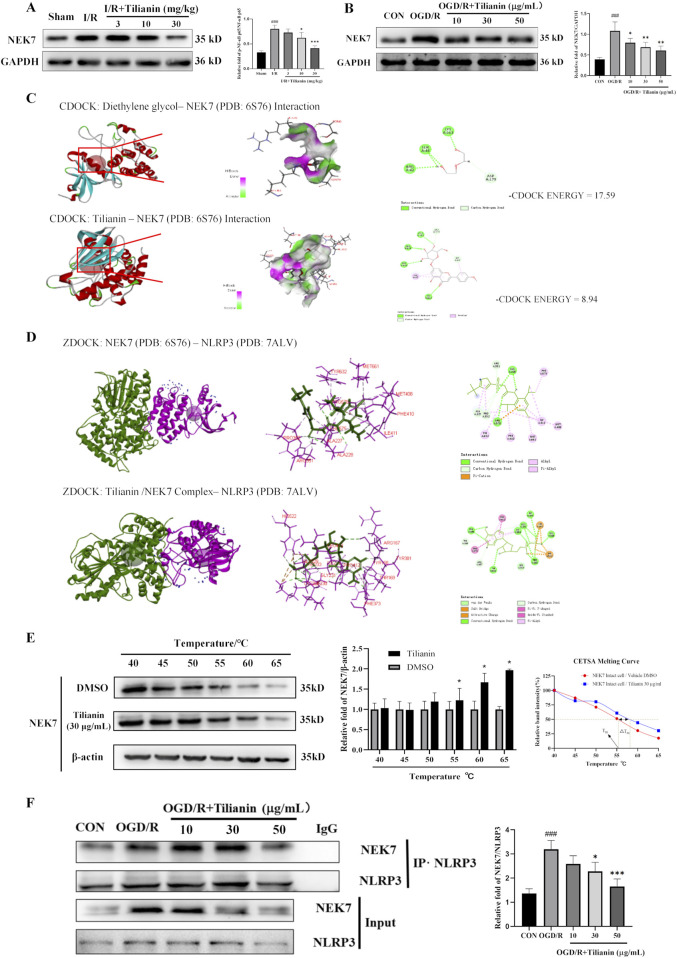
Tilianin lowered the level of NEK7 and disrupted the interaction of NLRP3/NEK7. **(A, B)** Tilianin reduced the levels of NEK7 in rats and H9c2 cells. n = 4 per group. **(C)** The docking diagram and the -CDOCKER ENGERGY values of NEK7 and tilianin or Diethylene glycol as an original ligand. **(D)** The impact of tilianin on the ZDocking of NEK7 and NLRP3 (6S76: Crystal structure of human NEK7 from the PDB, purple, 7ALV: Crystal structure of human NLRP3 from the PDB, green); **(E)** CETSA assay demonstrated the specific binding of tilianin to NEK7. The levels of NEK7 in tilianin-treated H9c2 cells lysates under different temperature were determined by western blotting (left and medium panel). The right panel displays the melting curve of NEK7. n = 3 per group. **(F)** Co-IP assay showed that tilianin cut down the interaction between NLRP3 and NEK7 in H9c2 cells. ##*P* < 0.01, ###*P* < 0.005 vs. sham or control group. *P < 0.05, **P< 0.01, ***P < 0.001 vs. I/R or OGD/R group.

It was indicated that tilianin possessed significant effects on inhibiting NLRP3 inflammasome in MIRI based on the results above. NEK7 has been identified as a pivotal role in the activation of the NLRP3 inflammasome and the induction of pyroptosis. Elevated levels of NEK7 were detected in both MIRI rats and OGD/R-treated H9c2 cells (Figures 6A, B). However, a marked reduction in NEK7 expression was observed upon administration of tilianin at the highest concentration of 50 μg/mL. CDOCKER ENERGY served as a metric to assess the interaction between tilianin and NEK7. The maximum value of -CDOCKER ENERGY generated by tilianin docking with NEK7 was 8.94, marginally lower than Diethylene glycol, suggesting a potential binding affinity of tilianin for NEK7 (Figure 6C). Tilianin could form Pi-Alkyl, conventional hydrogen bonds, and carbon hydrogen bonds with the amino acid residues of NEK7. At the same time, ZDOCK diagrams highlighted the optimal docking orientations and interfaces for both the NEK7-NLRP3 complex and the Tilianin/NEK7-NLRP3 complex (Figure 6D). The protein interface analysis showed the reduction of Pi interaction and hydrogen bonds, with the ligand contact surface area dropping to 293.77 Å and receptor contact surface area to 261.94 Å in Tilianin/NEK7-NLRP3 complex, compared with the NEK7-NLRP3 complex (Table 1). The CETSA assay demonstrated that as the temperature increases, the band intensity of NEK7 in the DMSO group progressively diminishes, indicating a reduction in the soluble NEK7 protein. In contrast, the protein contents of NEK7 at temperatures of 55, 60, and 65°C are significantly elevated after 30 μg/mL tilianin treatment, which is accompanied by a rightward shift in the melting curve (Figure 6E). This finding suggests a notable enhancement in the thermal stability of the NEK7 protein following tilianin treatment, implying the direct binding of tilianin to NEK7. Furthermore, co-IP assay was employed to elucidate the impact of tilianin on the interaction between the NLRP3 and NEK7. Western blot analysis revealed the enhanced interactions between NEK7 and NLRP3 following OGD/R when compared to the control group, but tilianin treatment resulted in a significant reduction in their interactions (Figure 6F). The findings indicate that tilianin significantly impeded the interaction between NLRP3 and NEK7.

**TABLE 1:** Protein interface analysis.

	ZDock score	Total Pi interaction	Total hydrogen bonds	Total salt bridges	Ligand contact surface area	Ligand Polar contact surface area	Ligand nonpolar contact surface area	Receptor contact surface area	Receptor Polar contact surface area	Receptor nonpolar contact surface area
6S76-7ALV	20.3	13	29	2	565.02	330.11	234.92	527.59	180.81	346.78
Tilianin/6S76-7ALV	19.6	6	22	2	293.77	224.18	69.597	261.94	90.212	171.73

Note: 6S76, Crystal structure of human NEK7 from the PDB; 7ALV, Crystal structure of human NLRP3 from the PDB.

## 4 Discussion

The present study elucidated the anti-inflammatory role of tilianin against MIRI and explored its underlying mechanisms. Tilianin exerted its anti-inflammatory effects by inhibiting the expression of NLRP3 and pro-inflammatory cytokines through the suppression of TLR4/NF-κB signaling pathway. Additionally, the interaction between NEK7 and NLRP3 also mediated the anti-inflammatory activities of tilianin in the context of MIRI.

Myocardial ischemia-reperfusion injury is a critical issue in cardiovascular disease management, involving complex pathophysiological processes, such as inflammation. The emerging evidence on tilianin suggested its anti-inflammatory activities to mitigating MIRI. Tilianin suppressed LPS-induced inflammatory responses in macrophages and inhibited TNF-α-induced proliferation and migration of vascular smooth muscle cells (Chen et al., 2022; Dong et al., 2022). Tilianin also inhibited CaMKII-dependent apoptotic pathway and the JNK/NF-κB inflammation pathway to exert cardioprotective effects against MIRI (Jiang et al., 2020a). Our data demonstrated that tilianin improved cardiac function, reduced infarct size, and attenuated the inflammation in rats undergoing MIRI. Additionally, the bioinformatics analysis highlighted the therapeutic potential of tilianin in MIRI and suggested its notable impacts on inflammatory regulation related pathways, such as NOD-like receptor signaling pathway.

Inspired by tilianin’s remarkable protective effects in MIRI, we exploited cytokines detecting assays and a quantitative proteomics study to probe its underlying mechanisms, revealing that tilianin exerted significant effects on inflammation regulation. It is generally recognized that TLR4/NF-κB signaling pathway is implicated in exacerbating the inflammatory response during MIRI, leading to increased cell damage and inflammatory cytokine production (Sun et al., 2016). Here, we found that tilianin effectively inhibited the phosphorylation of NF-κB p65, along with the level of NLRP3 in rats undergoing MIRI. Combined with the downregulation of IL-18 and IL-1β, it was implied that the NLRP3 inflammasome was inhibited by tilianin treatment in MIRI rats.

NLRP3 inflammasome is a critical component in the inflammatory response and plays a pivotal role in the development of MIRI (Chen et al., 2021; Toldo et al., 2022). It has been reported that the activation of the NLRP3 inflammasome is typically a two-step process: priming and activation. During priming, TLR4 detects endogenous danger signals and pathogen-associated molecular patterns and initiate immune response, triggering the phosphorylation of NF-κB which induces the expression of NLRP3 and pro-inflammatory cytokines, including IL-18 and IL-1β (Toldo et al., 2018; Rauf et al., 2019). Given the significant role of the NLRP3 inflammasome in the progression of MIRI, numerous inhibitors of the NLRP3 inflammasome have entered clinical trials. For instance, dapansutrile, a selective NLRP3 inflammasome inhibitor, has demonstrated both safety and efficacy in reducing joint pain and exhibited great potential in treating heart failure in a phase 1B trial (Klück et al., 2020; Wohlford et al., 2020). Additionally, DMF890, another small molecule NLRP3 inflammasome inhibitor, is currently in a phase 2 trial for coronary heart disease (Leu et al., 2023). Natural products are also acknowledged as valuable resources in cardiovascular diseases treatment, with some extracts found to inhibit NLRP3 inflammasome. A study showed that puerarin’s cardioprotective effects may be mediated via the SIRT1/NF-κB pathway and inhibition of the NLRP3 inflammasome (Wang Z. K. et al., 2020). Hence, we exploited OGD/R H9c2 cells to further investigate the effects of tilianin. The results were consistent with those in MIRI rats, which underscored that TLR4/NF-κB was inhibited by tilianin. It was worth noting that tilianin might also exert an inhibitory effect on the activation of the NLRP3 inflammasome, evidenced by the downregulation of cleaved caspase-1 and the reduction of ASC specks in H9c2 subjected to OGD/R. In this study, it remains uncertain whether NF-κB and NLRP3 are direct targets of tilianin or if they merely serve as downstream effectors. The interconnection of NF-κB and NLRP3 in inflammation is indeed pivotal. Utilizing specific agonists or antagonists for these proteins may yield valuable insights. However, it must be considered that these interventions could cause more confound responses and may not exclusively validate tilianin’s effects.

**FIGURE 7:**
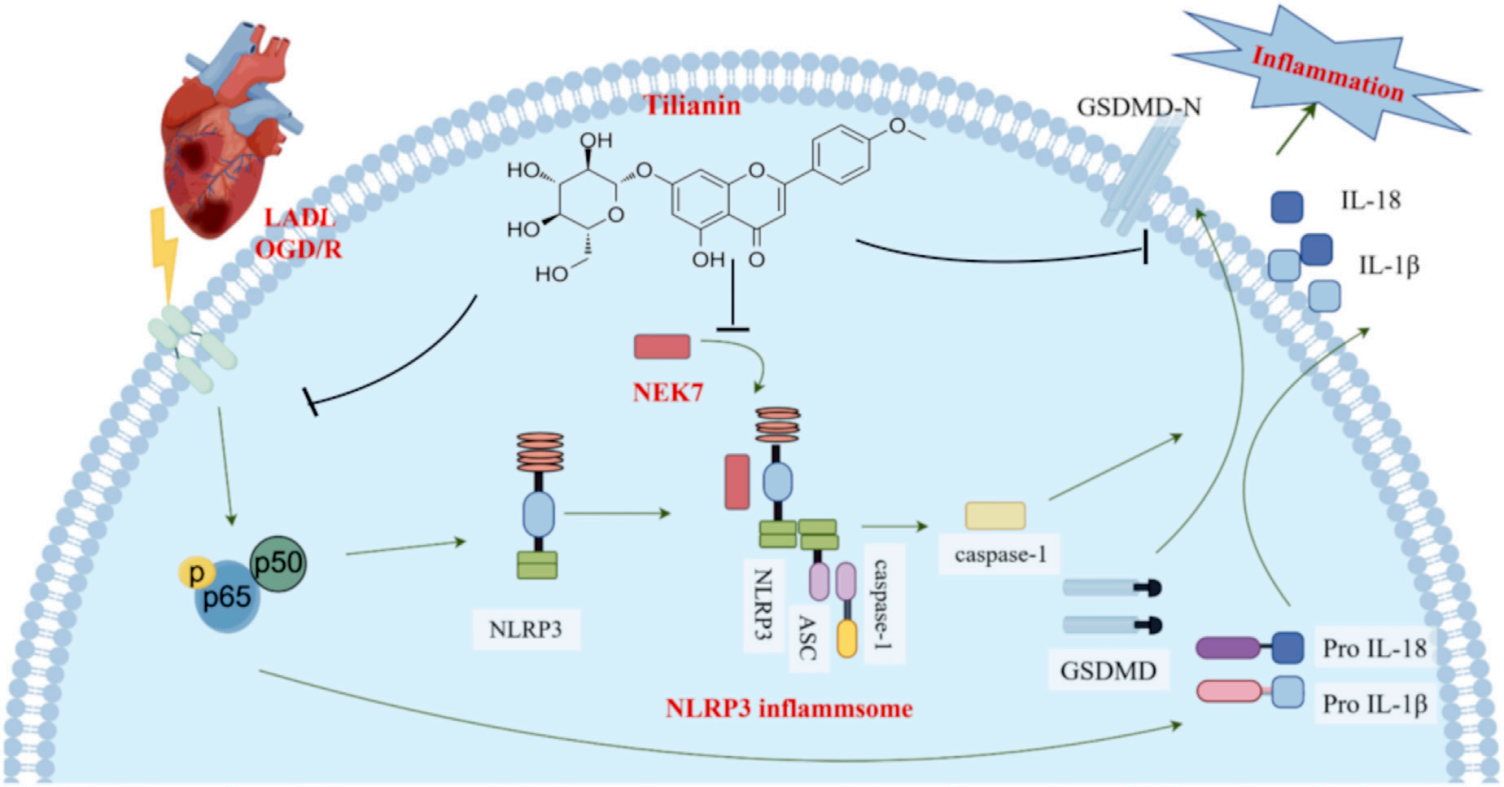
Tilianin suppressed NLRP3 inflammasome activation in myocardial ischemia/reperfusion injury via inhibition of TLR4/NF-κB and NEK7/NLRP3. When cardiomyocytes are exposed to LAD ligation (LADL) or OGD/R treatment, the TLR4/NF-κB pathway is activated, which increases the expression of NLRP3, pro-IL-18, and pro-IL-1β. Subsequently, NEK7 acts as a pivotal mediator in the activation of the NLRP3 inflammasome by binding to NLRP3 and facilitating the formation of the NLRP3 inflammasome complex, which comprises NLRP3, ASC, and pro-caspase-1. Caspase-1 is activated, promoting the release of GSDMD-N, IL-18, and IL-1β, leading to an inflammatory response and cell death. However, by effectively inhibiting the phosphorylation of NF-κB and disrupting the NEK7/NLRP3 interface, tilianin suppresses the activation of the NLRP3 inflammasome, reducing caspase-1 activation and ultimately decreasing the release of cleaved GSDMD, IL-1β, and IL-18, thus mitigating the inflammatory responses and minimizing myocardial ischemia-reperfusion injury (MIRI).

Upon encountering some stimuli, NLRP3 recruits ASC and pro-caspase-1 to form the NLRP3 inflammasome complex, leading to the auto-cleavage and activation of caspase-1 (He et al., 2016). Caspase-1 is responsible for converting pro-IL-1β and pro-IL-18 into IL-1β and IL-18, and for the cleavage of GSDMD, which ultimately results in the formation of pores in the cell membrane, triggering inflammation and cell death (He et al., 2015; Kelley et al., 2019). Recent findings highlight the essential role of NEK7 in the activation of the NLRP3 inflammasome (Shi et al., 2016). NEK7 is a serine/threonine kinase in the Never in Mitosis Gene A (NIMA) family and is involved in various cellular processes. It is reported that the C-terminal lobe of NEK7 nestles against the LRR and NACHT domains of human NLRP3 (Sharif et al., 2019). This combination promotes the conformational change of NLRP3, providing conditions for its subsequent self-polymerization which is required for NLRP3 inflammasome assembly and activation (He et al., 2016). In addition, a genome-wide CRISPR (clustered regularly interspaced short palindromic repeats) screen identifies NEK7 as an essential component of NLRP3 inflammasome activation (Schmid-Burgk et al., 2016). At the same time, inhibitors targeting the NEK7-NLRP3 interaction, such as artemisinin and berberine, have shown promise in mitigating inflammation by interfering with the NLRP3 activation pathway, thus providing therapeutic potential (Kim et al., 2019; Zeng et al., 2020). Therefore, targeting the NLRP3-NEK7 interaction directly to inhibit NLRP3 activation emerges as a feasible strategy to treat MIRI. In this study, our findings suggest that tilianin plays a significant role in directly inhibiting this NLRP3-NEK7 interaction. CETSA results showed that the thermal stability was significantly improved after treatment with 30 mg/kg tilianin, indicating that tilianin can indeed specifically target NEK7 protein. Moreover, co-IP assay and molecular docking results demonstrated that tilianin cut down the level of NLRP3-NEK7 complex. Also, we acknowledge that the reduced interaction between NLRP3 and NEK7 may be influenced by lower NEK7 levels. It is indeed noteworthy that the significant inhibition of NEK7 protein expression by tilianin occurs primarily at the highest concentration, i.e., 50 μg/mL, which may suggest that this effect represents a secondary mechanism of action. Future studies will be essential to clarify the underlying processes by which tilianin reduces NEK7 expression level. Our findings elucidated that tilianin disrupted the NLRP3-NEK7 interaction, proposing a supplemental mechanism by which tilianin treatment could mitigate NLRP3 inflammasome activation and alleviate myocardial ischemia/reperfusion injury (Figure 7).

In conclusion, this study demonstrated that the TLR4/NF-κB/NLRP3 pathway is involved in the anti-inflammatory and anti-myocardial ischemia/reperfusion injury (MIRI) properties of tilianin. Furthermore, tilianin can disrupt the NEK7/NLRP3 interface, thereby inhibiting the activation of the NLRP3 inflammasome. Overall, tilianin suppresses NLRP3 inflammasome activation in myocardial ischemia/reperfusion injury by inhibiting the TLR4/NF-κB and NEK7/NLRP3 pathways.

## Data Availability

The raw data supporting the conclusions of this article will be made available by the authors, without undue reservation.

## Abbreviations

ASC, apoptosis associated speck like protein containing a CARD; AST, aspartate aminotransferase; cTnT, cardiac troponin T; CETSA, cellular thermal shift assay; DMEM, Dulbecco’s modified Eagle’s medium; FBS, fetal bovine serum; GSDMD, gasdermin D; HE, hematoxylin-eosin; IL-18, interleukin-18; IL-1β, interleukin-1β; LDH, lactate dehydrogenase; LVEDd, left ventricular end-diastolic diameter; LVED, left ventricular end-systolic diameter; LVEF, left ventricular ejection fraction; LVFS, left ventricular fractional shortening; MIRI, myocardial ischemia-reperfusion injury; NEK7, NIMA-related kinase 7; NLRP3, nucleotide-binding domain and leucine-rich repeat-containing family pyrin domain-containing protein 3; OGD/R, oxygen-glucose deprivation/reoxygenation; PBS, phosphate buffer solution; SDS-PAGE, sodium dodecyl sulfate-polyacrylamide gel electrophoresis; TNF-α, tumor necrosis factor-α; TTC, 2,3,5-triphenyl-tetrazolium chloride; TUNEL, TdT-mediated dUTP Nick-End Labeling.
